# On-farm colorimetric detection of *Pasteurella multocida*, *Mannheimia haemolytica*, and *Histophilus somni* in crude bovine nasal samples

**DOI:** 10.1186/s13567-021-00997-9

**Published:** 2021-10-02

**Authors:** Ana Pascual-Garrigos, Murali Kannan Maruthamuthu, Aaron Ault, Josiah Levi Davidson, Grigorii Rudakov, Deepti Pillai, Jennifer Koziol, Jon P. Schoonmaker, Timothy Johnson, Mohit S. Verma

**Affiliations:** 1 Department of Agricultural and Biological Engineering, Purdue University, 225 S University Street, West Lafayette, IN 47907 USA; 2 Department of Biochemistry, Purdue University, 175 South University Street, West Lafayette, IN 47906 USA; 3 Birck Nanotechnology Center, Purdue University, 1205 W State St, West Lafayette, IN 47907 USA; 4 School of Electrical and Computer Engineering, Purdue University, 465 Northwestern Avenue, West Lafayette, IN 47907 USA; 5 Weldon School of Biomedical Engineering, Purdue University, 206 South Martin Jischke Drive, West Lafayette, IN 47907 USA; 6 Department of Comparative Pathobiology, Purdue University, 625 Harrison Street, West Lafayette, IN 47907 USA; 7 School of Veterinary Medicine, Texas Tech University, 7671 Evans Drive , Amarillo , TX 79106 USA; 8 Department of Animal Sciences, Purdue University, 270 S Russell Street, West Lafayette, IN 47907 USA

**Keywords:** diagnostics, bovine respiratory disease, loop-mediated isothermal amplification, pen-side

## Abstract

This work modifies a loop-mediated isothermal amplification (LAMP) assay to detect the bovine respiratory disease (BRD) bacterial pathogens *Pasteurella multocida*, *Mannheimia haemolytica*, and *Histophilus somni* in a colorimetric format on a farm. BRD causes a significant health and economic burden worldwide that partially stems from the challenges involved in determining the pathogens causing the disease. Methods such as polymerase chain reaction (PCR) have the potential to identify the causative pathogens but require lab equipment and extensive sample processing making the process lengthy and expensive. To combat this limitation, LAMP allows accurate pathogen detection in unprocessed samples by the naked eye allowing for potentially faster and more precise diagnostics on the farm. The assay developed here offers 66.7–100% analytical sensitivity, and 100% analytical specificity (using contrived samples) while providing 60–100% concordance with PCR results when tested on five steers in a feedlot. The use of a consumer-grade water bath enabled on-farm execution by collecting a nasal swab from cattle and provided a colorimetric result within 60 min. Such an assay holds the potential to provide rapid pen-side diagnostics to cattle producers and veterinarians.

**Supplementary Information:**

The online version contains supplementary material available at 10.1186/s13567-021-00997-9.

## Introduction

Bovine respiratory disease (BRD) is known to be the most common cause of morbidity and mortality in cattle affecting 16% of all beef cattle [1] and causing up to 75% feedlot morbidity in North America [2]. It is also estimated to cost up to $900 million annually in the beef industry alone [3]. This economic burden includes animal weight loss, labor expenses, pharmaceutical costs, and deaths [4]. Rapid on-farm diagnostics have the potential to reduce this economic burden.

BRD is an umbrella term used to describe a condition caused by bacteria, viruses, or co-infection [5, 6]. Generally, it is detected by observing clinical signs such as nasal discharge, depression, anorexia, cough, and fever [4]. However, these clinical signs are insufficient for determining the underlying causative pathogen. Currently, one method for determining which pathogen is causing BRD involves taking a nasal swab sample from the suspected animal and sending it to a diagnostic laboratory, where tests are carried out to identify pathogens [7].

Some of the existing methods used for identifying the pathogen include culturing, enzyme-linked immunosorbent assays (ELISA), electron microscopy, immunohistochemistry, microarrays, and quantitative PCR (qPCR) detection [7, 8]. Unfortunately, tests of this nature often require specialized equipment, extensive sample preparation, and trained scientists making the process costly and lengthy. A mainstay of clinical therapy is the use of broad-spectrum antibiotics, which can have high failure rates [9]. Antibiotic therapy can fail for several reasons including misdiagnosis, inappropriate drug selection, inappropriate administration rate, dehydration, etc. At the same time, the use of antibiotics is claimed to be the leading cause of antimicrobial resistance, which makes the problem worse over time [10]. Thus, diagnostics that identify the pathogen and potentially their antibiotic resistance can help improve treatment by guiding towards targeted antibiotic use.

Unlike the lab-based methods mentioned above, isothermal DNA amplification methods such as recombinase polymerase amplification (RPA) [11] and loop-mediated isothermal amplification (LAMP) [12–14] have recently been shown to accurately detect BRD pathogens directly from nasopharyngeal or nasal swabs used on cattle [15, 16]. Like the versatile PCR, LAMP can detect sections of DNA found in infectious pathogens. However, it can do so in under an hour and without the need of a thermal cycler [17] making it more field-friendly for use in non-lab spaces such as farms.

We have recently designed a LAMP assay to detect the presence of *Pasteurella multocida*, *Mannheimia haemolytica*, and *Histophilus somni* from bovine nasal samples using a fluorescence reporter with 89% analytical specificity and 99% analytical sensitivity [16]. In the current work, we report a colorimetric assay that can be conducted on the farm to detect these bacterial pathogens. The colorimetric assay has a limit of detection of 1250 copies of DNA per reaction, an analytical specificity of 100%, and an analytical sensitivity in the range of 66.7–100% (when measured using contrived samples). The color change is visible to the naked eye and quantifiable using a camera. We illustrate the functionality of this assay on a feedlot by using a simple water bath based on consumer-grade precision cookers and conducting the assay on nasal samples obtained from five steers. The on-farm results are in 60–100% agreement with PCR assays conducted in the lab on the same samples.

## Materials and methods

### Bacterial isolates and mucus sample collection

Glycerol stocks of *P. multocida*, *M. haemolytica*, and *H. somni* isolates were obtained from Purdue University’s Indiana Animal Disease Diagnostic Laboratory (ADDL), as previously described [16]. These isolates were originally cultured by ADDL as a part of routine diagnostic testing from lung/nasopharyngeal sample submissions and identified using matrix-assisted laser desorption/ionization time-of-flight mass spectrometry (MALDI-TOF MS) analysis. Mucus samples were obtained from steers (*n* = 5) approximately 12 months of age and 600 lbs. in weight that had not been given antibiotics for at least 100 days at a feedlot in Indiana (Purdue Animal Care and Use Committee Approval #1906001911) using rayon-tipped sterile double swabs designed for general specimen laboratory use (BD 220135, Becton, Dickinson, and Company, Franklin Lakes, NJ, USA). Each animal was restrained in a livestock handling chute and the animal head was restrained to minimize movement. The nostrils were wiped with paper towels to remove excess mucus. One double swab was inserted into both nostrils sequentially at a depth of approximately 5 cm. The swabs were swirled in tubes with 200 µL of DNA-free water and aliquoted for use in LAMP reactions for both on-farm and in-lab settings.

### Bacterial DNA isolation and quantification

*P. multocida*, *M. haemolytica*, and *H. somni* were isolated and final DNA concentrations were quantified according to Mohan et al. [16]. Specifically, all pathogens were streaked on tryptic soy agar plates supplemented with defibrinated sheep blood (blood agar). *P. multocida* and *M. haemolytica* were incubated aerobically at 37 °C for 16–18 h while *H. somni* was incubated in a 5% CO_2_ atmosphere at 37 °C for 2–3 days by using BD GasPak™ EZ container systems (BD 260672) with BD BBL™ CO_2_ gas generators (BD 260679). Individual colonies of each bacterial species were picked from the blood agar plates. *P. multocida* and *M. haemolytica* were inoculated into brain–heart infusion (BHI) broth and *H. somni* was inoculated into tryptic soy broth (TSB). All were incubated in the same conditions as the plates.

DNA extraction of each pathogen was carried out by taking 2 mL of saturated liquid culture and following the PureLink™ Genomic DNA Mini Kit (Catalog #K182002, Invitrogen, Waltham, MA, USA) procedure. Final DNA concentrations (ng/µL) of eluted extracts were measured using the Quant-iT PicoGreen dsDNA Assay Kit (Invitrogen P11496).

### Colorimetric quantitative LAMP assay (qLAMP)

The colorimetric assay was conducted by modifying the previously published procedure [16]. Specifically, in the colorimetric assay, the New England Biolabs’ Warmstart Colorimetric LAMP 2 × Master Mix was used. The mix was coupled with Antarctic Thermolabile uracil DNA glycosylase (UDG) and deoxyuridine triphosphate (dUTP) to minimize carryover contamination throughout the experiment. In-house validation experiments have confirmed that UDG/dUTP does not affect reaction performance at the concentration used. The LAMP solution comprised 12.5 µL of this mix (40 mM Tris–HCl, 20 mM (NH_4_)_2_SO_4_, 100 mM KCl, 16 mM MgSO_4_, 2.8 mM dNTPs,0.28 µM dUTP, 0.64 U/µL Warmstart^®^ Bst 2.0 DNA polymerase, 0.6 U/µL Warmstart^®^ Reverse Transcriptase [RTx], 4 × 10^–4^ U/µL Antarctic Thermolabile UDG, 200 mM Phenol red, 0.2% Tween 20, pH 8.8@25 °C) (Catalog # M1800L, New England Biolabs, Ipswich, MA, USA), 2.5 µL of a 10 × LAMP primer mixture (10× concentration: 2 µM F3, 2 µM B3, 4 µM LF, 4 µM LB, 16 µM FIP, 16 µM BIP), 5 µL of DNA-free water, and 5 µL of DNA or mucus containing solution. Reactions were pipetted into wells of clear 96-well FrameStar^®^ skirted flat optical bottom PCR plates (Catalog #1149V67, Thomas Scientific, Swedesboro, NJ, USA). Wells were sealed with adhesive PCR plate seals (Catalog #AB-0558, Thermo Fisher Scientific Waltham, MA, USA) and inserted into a CLARIOstar Plus (BMG Labtech Cary, NC, USA), a multi-mode plate reader with temperature control, for real-time colorimetric measurement. Spectra were collected from 350 to 750 nm with a step size of 5 nm for 60 cycles lasting approximately 60 s each. Reaction plates were incubated at 65 °C using the CLARIOstar Plus.

Each step in LAMP preparation (primer addition, template DNA loading, and reaction incubation/measurement) was conducted in separate lab spaces in order to minimize false positives due to amplicon aerosol contamination. To further reduce contamination, RNase AWAY™ Surface Decontaminant (Thermo Fisher Scientific 14-754-34) was thoroughly applied to all working surfaces and lab gloves before and after use and wiped completely with Kimwipes to prevent residue formation.

### Data analysis

Absorbance measurements for each minute at 430, 560, and 620 nm wavelengths were extracted, and the data were normalized using the formula (Equation 1):

1 $${\text{Colorimetric}}\,{\text{absorbance}}\,{\text{ratio}} = \frac{{{\text{Absorbance at }}\,430{\text{ nm}} - {\text{Absorbance at }}\,620{\text{ nm}}}}{{{\text{Absorbance at }}\,520{\text{ nm}} - {\text{Absorbance at }}\,620{\text{ nm}}}}$$

The absorbance at 620 nm was used as a baseline, and the 430 nm and 520 nm wavelengths were used to mark the change in color of phenol red from red to yellow. The resulting ratios were plotted against time in Microsoft Excel.

### Colorimetric threshold

A one-to-one mixture of pH 7.2 phosphate-buffered saline (PBS, Thermo Fisher Scientific 20012050) and pH 8.5 Tris–HCl (Catalog #SD8141, Bio Basic Amherst, NY, USA) was prepared. Using 0.1 M NaOH and 0.1 M HCl, the solution was adjusted to range between pH 6.0 and 8.0 with increments of approximately 0.2 pH units. 5 µL of the buffer solution were added to 12.5 µL of Warmstart^®^ Colorimetric LAMP 2 × Master Mix and 7.5 µL of DNA-free water. Each condition was added to a 96-well FrameStar^®^ skirted flat optical bottom PCR plate in triplicate, sealed with a PCR film, and inserted into the CLARIOstar Plus to obtain measurements for three minutes. Data were analyzed as explained above and the values at each minute were averaged. From plotting the data, a colorimetric absorbance ratio was selected as the threshold according to the color changes observed so that colorimetric absorbance ratios above the threshold were considered positive and colorimetric absorbance ratios below the threshold were considered negative.

An image of the plate was taken using an Epson Perfection V800 Photo scanner (Catalog #B11B223201, Amazon, Seattle, WA, USA) with settings set to professional mode, 48-bit color image type, and 720 dpi resolution. The image of the sample closest to the threshold value was processed using ImageJ to find the RGB values. Those RGB values were in turn used to calculate Hue Saturation Values (HSV) values. The Hue threshold was used to determine positive (above Hue threshold) versus negative (below Hue threshold) results in other assays.

### Primer screening and limit of detection (LOD)

Colorimetric absorbance ratios were obtained from qLAMP experiments using a 2 × DNA dilution factor (10 000–78.125 copies of DNA per reaction). Note that we are including the fraction of copies simply to indicate the dilution factor, the actual number of copies in a reaction would be rounded to the nearest whole number. All primer sets in Additional file 1 were tested and each concentration of the template included three replicates for each primer set. DNA-free water was used as a control (no-template control, NTC).

Primer sets were scored by annotating the number of sufficient amplification reactions—defined as any replicate whose colorimetric absorbance ratio at 60 min was greater than 3.0—for each template concentration [including (NTC)] for each primer set. Any replicate that was deemed as sufficient amplification in the NTC was designated as a false positive. Any missing data for an entire template concentration was set at a constant value equal to the maximum colorimetric absorbance ratio observed across all primer sets at all concentrations. In contrast, any missing data for any given time point was filled with the value of the previous time point.

Primer sets were scored by first calculating the maximum colorimetric absorbance ratio and reaction time for each replicate at each template concentration (excluding NTC) for a given primer set. The average and standard deviation of these values were then calculated for each template concentration for a given primer set. Reaction time was defined as the first time point at which the absorbance ratio was greater than 3.0. For each primer set, the average of each one of these four metrics (average and standard deviation of maximum intensity and reaction time) was calculated across all template concentrations to assign a primer set metric (e.g., primer set average maximum colorimetric absorbance ratio). The LOD for each concentration was then calculated as the minimum template concentration where all replicates sufficiently amplify and all replicates of template concentrations above this minimum template concentration also sufficiently amplify. For cases where all replicates for all tested template concentrations amplified, the LOD was set at the lowest non-zero template concentration if there were less than three false positives. If all NTC reactions amplified (i.e., three false positives) or no replicates amplified at any template concentration, the LOD was set at −1.

For the overall scoring of primer sets, ineligible primer sets (as designated by an LOD of −1) were automatically assigned an overall score of 0 and withdrawn from further scoring. All eligible primer sets were then assigned a weighted overall score, S_k_, for a primer set, k, using the following expression:

$$S_{k} = w_{{\overline{I}}} \cdot \left( {1 - \frac{{\max \left( {\overline{I}} \right) - I_{k} }}{{Range\left( {\overline{I}} \right)}}} \right) + \mathop \sum \limits_{n} w_{n} \cdot \left( {1 - \frac{{\min \left( n \right) - n_{k} }}{Range\left( n \right)}} \right)$$

$$n \in \left( {\sigma \left( I \right), \,\overline{{t_{rxn} }} ,\,\sigma \left( {t_{rxn} } \right), \,LOD,\,FP } \right)$$

$$w_{n} = \left[ {\begin{array}{*{20}c} {35} \\ 5 \\ {30} \\ 5 \\ 5 \\ {20} \\ \end{array} } \right]for \begin{array}{*{20}c} {\overline{I}} \\ {\sigma \left( I \right)} \\ {\overline{{t_{rxn} }} } \\ {\sigma \left( {t_{rxn} } \right)} \\ {LOD} \\ {FP} \\ \end{array}$$

where $\overline{I},\sigma \left( I \right), \overline{{t_{rxn} }} ,\sigma \left( {t_{rxn} } \right), LOD,FP$ is the set average maximum colorimetric absorbance ratio, set standard deviation of the maximum colorimetric absorbance ratio, set average reaction time, set standard deviation of the reaction time, set LOD, and the number of false positives for a given primer set, respectively. The range defined above is the maximum value minus the minimum value for a given set metric across all eligible primer sets. If the range for a given set metric was 0 (i.e., all primer sets had the same value), that set was given the full weight allotted for that set metric.

Primers with the highest scores were selected as the best primer sets to detect the bacteria of interest using a python script (Additional file 2). The worst LOD for the three selected primers was set as the LOD to be used in other experiments. Images of the plates were taken at 0 and 60 min using the Epson Perfection V800 Photo scanner.

### Combinatorial experiment with one, two, or three species spiked into water

Colorimetric qLAMP assays were performed for 60 min using 1250 copies per reaction of gDNA of one, two, and/or three bacteria *P. multocida*, *M. haemolytica*, and *H. somni* in the same reaction. Each condition was repeated nine times in nine separate wells of 96-well plates. Images of the plates were taken at 0 and 60 min using the Epson Perfection V800 Photo scanner. Colorimetric absorbance ratios were calculated as explained above, and the resulting data were plotted against time. The finalized data were also analyzed in receiver operating characteristic (ROC) curves by using the colorimetric threshold previously determined and assessing positive versus negative reactions for each primer set. The highest number obtained from subtracting the false positive rate (Equation 2) from the true positive rate (Equation 3) was selected as the time threshold for that specific primer.

2 $${\text{False}}\,{\text{positive}}\,{\text{rate}}\,({\text{FPR}}) = \frac{{{\text{no}}{.}\,{\text{of}}\,{\text{false}}\,{\text{positives}}}}{{{\text{no}}{.}\,{\text{of}}\,{\text{false positives}} + {\text{no}}{.}\,{\text{of}}\,{\text{true}}\,{\text{negatives}}}}$$

3 $${\text{True}}\,{\text{positive}}\,{\text{rate}}\,({\text{TPR}}) = \frac{{{\text{no}}.{\text{ of true positives}}}}{{{\text{no}}.{\text{ of true positives }} + {\text{no}}.{\text{ of false negatives}}}}$$

### Precision cooker experiments (on-farm and in-lab)

LAMP reactions were prepared in individual domed PCR tubes (Thermo Fisher Scientific AB0337) using 12.5 µL New England Biolabs’ Warmstart^®^ Colorimetric LAMP 2 × Master Mix, 2.5 µL of primer mix, 5 µL of DNA free water, and 5 µL of mucus sample. An Anova Culinary AN500-US00 Sous Vide Precision Cooker (Amazon B08CF6Y4WF) was filled with water and set to 149 °F (65 °C). The temperature of the water was verified in the lab using an Hti HT-04 Thermal Imaging Camera (Additional file 3). The tubes were submerged in the water on the right side (the region with a relatively homogenous temperature of 65 °C) either by taping them to the inside of the precision cooker with heat-resistant 3/4-inch autoclave tape (Thermo Fisher Scientific 15904) or by using PCR tube holders designed and 3D-printed in-lab with a Formlabs Form 3B 3D printer (Formlabs, Somerville, MA, USA) using high-temperature resin v2 and 0.1 mm layer thickness (Additional files 4 and 5). The tubes were removed from the water after 60 min.

The experiment was performed in-lab using the usual procedures to avoid contamination (RNase AWAY™ spray, separation of lab spaces, etc.) and on-farm. For the on-farm experiment, the reagents were prepared in the lab, and the addition of mucus was done on-farm using a 0.5–10 µL single-channel pipette with no additional measures to avoid contamination (Additional file 6). The mucus addition on-farm happened no more than 30 min after extraction from the steers, while the mucus addition in the lab was done 4 days after collecting the samples (the samples being stored at −80 °C in the meantime). The samples were stored in water so that the test matrix would be similar in the lab and on the farm.

Images of the tubes were taken at 0 and 60 min. Images of the tubes in-lab were taken using the Epson Perfection V800 Photo scanner and images of tubes in-farm were taken using a Samsung Galaxy A50. All images obtained were adjusted by using the white balance tool on Adobe Lightroom to obtain a relatively uniform background. The RGB values of each solution were extracted at 60 min using ImageJ and Hue values were calculated to differentiate positive and negative results. Shadows and glows on the images were avoided during this process to increase the accuracy of the results. The Hue scale indicated on a color wheel from 0° to 360°. Red/pink color is around 0–15° and 345–360°, orange/yellow is around 30–60°. Since we set a Hue value of 35 as cut-off (higher than 35 is a positive reaction), the red/pink color on the high end (close to 360°) was simply set to 0 to avoid confusion.

When comparing the LAMP farm results with PCR, having 2 out of 3 LAMP reactions show the same result as PCR was considered agreement.

## Results

### LAMP colorimetric performance

**Figure 1:**
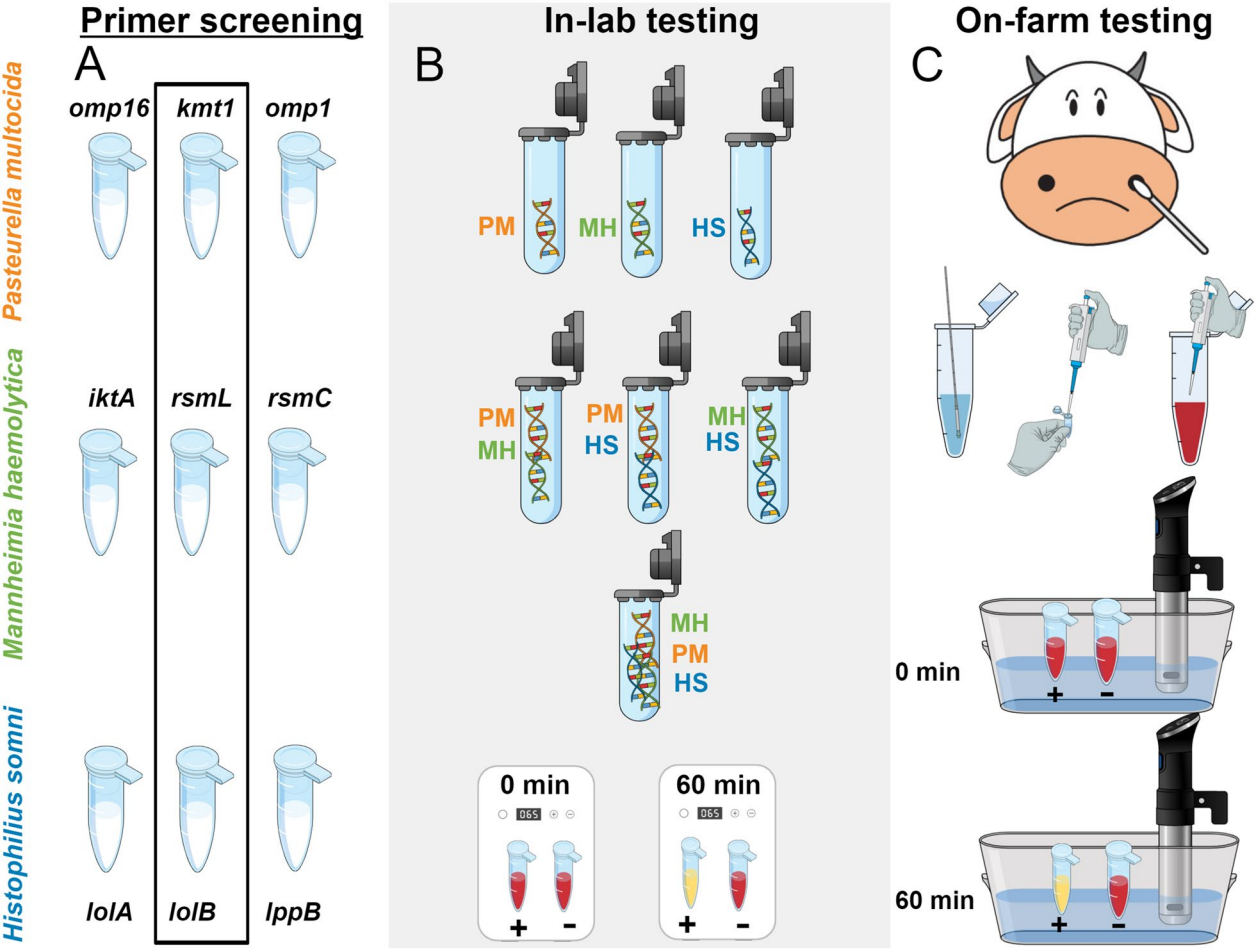
**Overall schematic of the workflow. A** Three different primers were screened through the limit of detection (LOD) study. The best-selected primers in each species were highlighted inside the black rectangle. **B** Several combinations of DNA were diluted in water and tested in the lab environment to study off-target behavior in pH-sensitive colorimetric reactions. **C** Loop-mediated isothermal amplification (LAMP) was conducted on-farm with a prepared colorimetric master-mix and later repeated in-lab. A precision cooker was used as a heating device to confirm the ability of our test in a resource-limited setting. PM, *Pasteurella multocida*; MH, *Mannheimia haemolytica*; HS, *Histophilus somni*.

**Figure 2:**
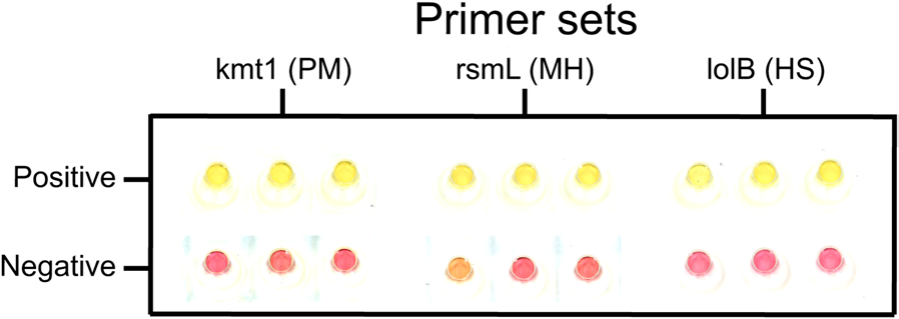
**Representative colorimetric results for positive and negative reactions.** Positives taken from quantitative loop-mediated isothermal amplification (qLAMP) reactions run with 10 000 copies of DNA per reaction and negatives taken from qLAMP reactions without DNA. Kmt1 primers were used to detect PM, rsmL primers were used to detect MH and lolB primers were used to detect HS. All samples were imaged at 60 min. Images were collected using an Epson Perfection V800 Photo scanner and the background was whitened using the ImageJ brightness/contrast setting. PM, *Pasteurella multocida*; MH, *Mannheimia haemolytica*; HS, *Histophilus somni*.

We evaluated the analytical sensitivity and specificity of primers designed to detect BRD pathogens as highlighted in Figure 1. A typical colorimetric response between positive and negative results is shown in Figure 2. We present the percent concordance between in-lab and on-farm LAMP as well as between PCR and on-farm LAMP using unprocessed mucus collected from steers (Table 1 and Additional file 7).

**Table 1:** **Concordance between experiments in-lab and on-farm, and between the precision cooker assay on-farm and polymerase chain reaction (PCR)**

Target pathogen	% Concordance: precision cooker on-farm vs. in-lab PCR (%)	% Concordance: precision cooker on-farm vs. in-lab (%)
*P. multocida*	100.0	83.3
*M. haemolytica*	60.0	66.7
*H. somni*	100.0	66.7

Between loop-mediated isothermal amplification (LAMP) and PCR, 2 out of 3 LAMP reactions with the same result as PCR was considered agreement.

### Determining a threshold for colorimetric visualization

**Figure 3:**
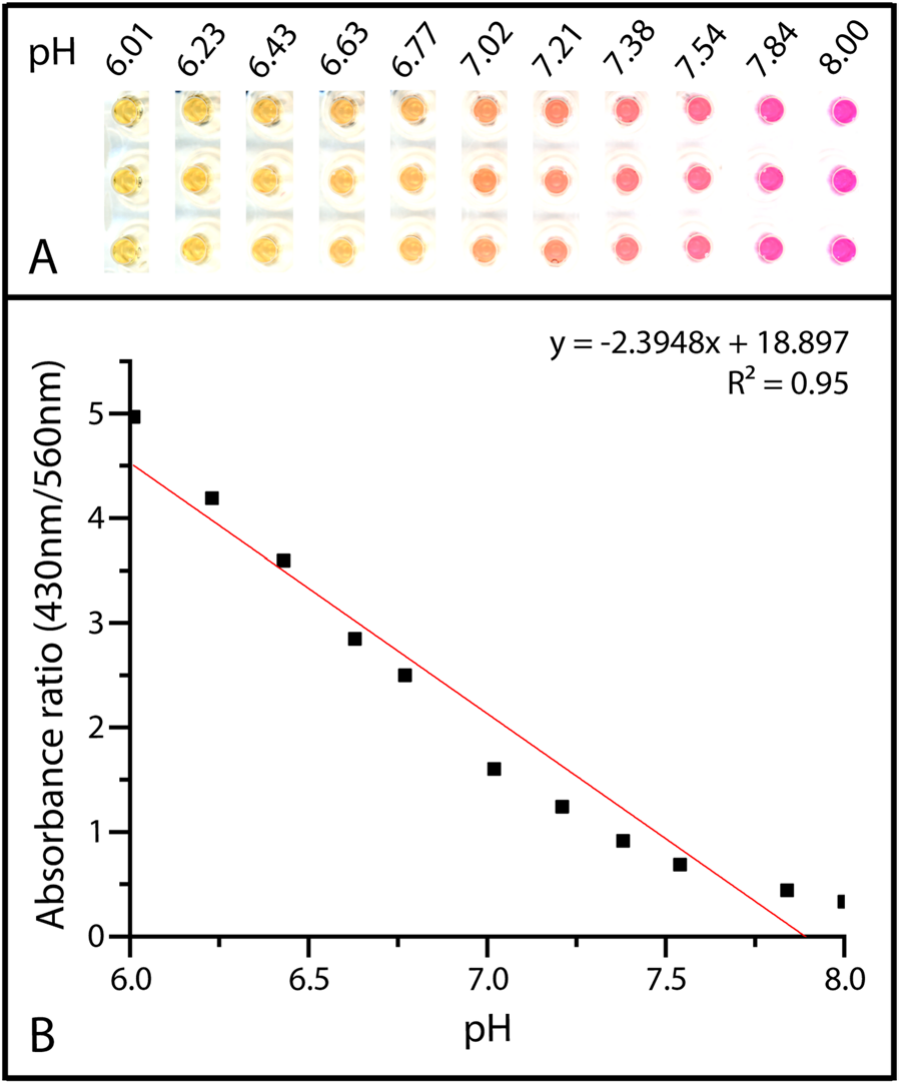
**Colorimetric gradient at increasing pH values.** Colorimetric detection of a 1:1 mixture of phosphate-buffered saline (PBS) and Tris–HCl buffer adjusted to pH 6.0–8.0 in approximately 0.2 pH intervals. **A** Image collected using the Epson Perfection V800 Photo scanner and background whitened using the ImageJ brightness/contrast setting. **B** Average colorimetric absorbance ratios from three cycles collected with the CLARIOstar Plus.

LAMP reaction reagents were set to increasing pH to visualize color change (Figure 3). From the colorimetric results, 3.0 was selected as the threshold for colorimetric absorbance ratio. The color of the LAMP reaction at pH 6.63 (which is the closest to the 3.0 threshold of all the samples tested) was used to set the Hue threshold at 35 since at this point, the color change is distinctly different from the starting reaction color around pH 7.5–8.

### Down selection of primers for BRD detection through quantitative LAMP and LOD experiments

From the primer screening carried out in Mohan et al. [16], the selected primers (Additional file 1) were narrowed down further to one per bacterial target. The optimal primers were selected based on calculated scores (Additional file 2) obtained from LOD colorimetric assays (Additional files 8, 9, 10). The primers with the highest scores were kmt1, rsmL, and lolB as highlighted in blue shaded data (Table 2). The worst limit of detection of the three selected primers, 1250 copies per reaction, was selected as the concentration used in later multiple isolate experiments.

**Table 2:**
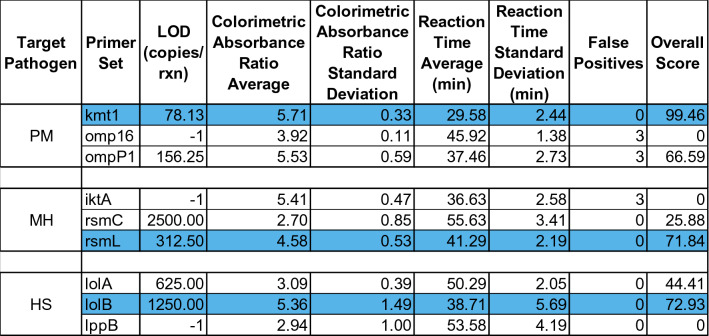
**Performance characteristics of all primer sets used for screening**

Higher overall score indicates better performance.

PM, *Pasteurella multocida*; MH, *Mannheimia haemolytica*; HS, *Histophilus somni*.

The data highlighted in blue represents the selected primer sets as determined by the highest score for the target pathogen.

### Combinatorial experiment with one, two, or three bacterial species spiked in water using phenol red

Colorimetric qLAMP reactions with the selected primer sets were performed to determine assay performance with mixed bacterial samples. The previously determined limit of detection, 1250 copies per reaction of gDNA, was spiked in for each pathogen being tested in a reaction. Pathogens were tested in pairs and all together to simulate the bacterial communities present in actual mucus samples. The kmt1 (PM) and lolB (HS) primer sets were able to amplify the corresponding genes regardless of the bacterial combination and showed minimal false-positive results. RsmL (MH) also showed a colorimetric difference in the presence versus the absence of *M. haemolytica* genes. However, it was not significant enough for the previously determined standards of 3.0 colorimetric absorbance threshold and 35 Hue value since some samples with target pathogenic DNA showed results below these values (Additional files 11 and 12).

**Figure 4:**
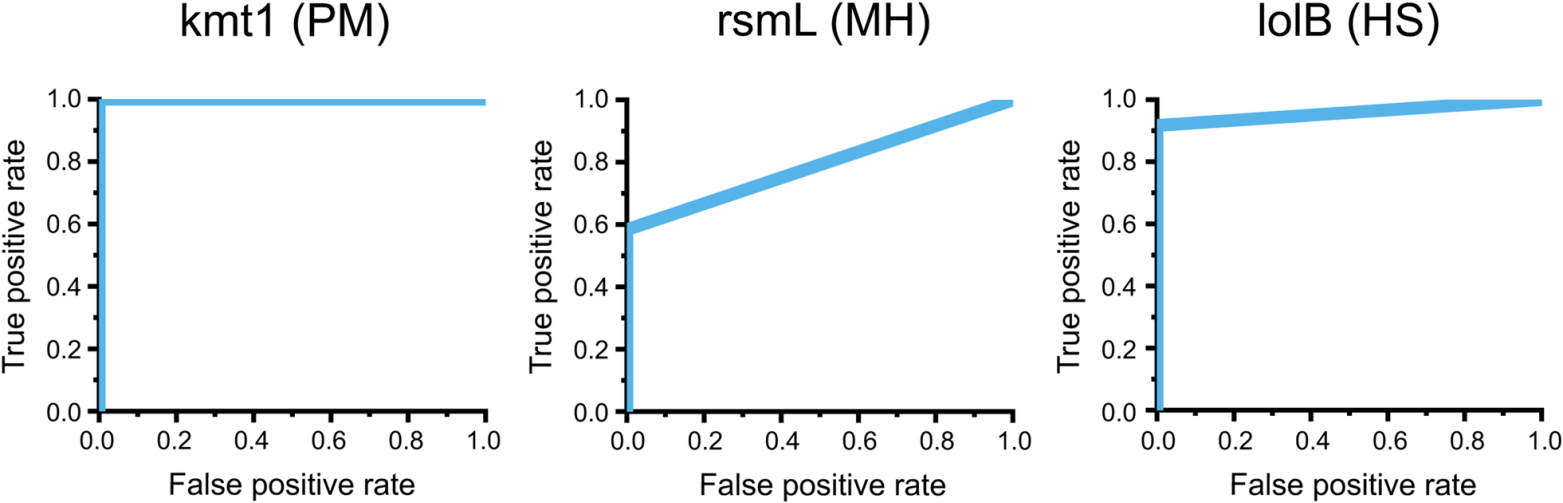
**Receiver operating characteristic (ROC) curves for each selected primer set.** The graphs illustrate the true-positive rate (TPR) and false-positive rate (FPR) of the quantitative loop-mediated isothermal amplification (qLAMP) assay for each primer using the combinations of bovine respiratory disease (BRD) bacteria presented in Additional files 11 and 12. TPR was calculated as TP/(TP + FN) and FPR was calculated as FP/(FP + TN). TP, true positive; FN, false negative; FP, false positive; TN, true negative; PM, *Pasteurella multocida*; MH, *Mannheimia haemolytica*; HS, *Histophilus somni*.

These results were analyzed further by generating ROC curves for each primer set. Here, the kmt1 and lolB primer sets show nearly perfect curves (analytical sensitivity of 100% (kmt1) and 91.67% (lolB); analytical specificity of 100% (kmt1) and 100% (lolB)), while rsmL does not. This indicates that rsmL does not work well (66.7% analytical sensitivity, 100% analytical specificity) and will need to be redesigned in future studies for improving analytical sensitivity (Figure 4). These curves were also used to identify a time threshold (i.e., if a reaction requires more than this threshold to obtain a colorimetric absorbance ratio of 3.0, it is considered a negative result) for each of the primer sets by finding the time throughout the 60-min period with the greatest difference between the true positive and false-positive rates. The selected time thresholds were: 41 min (kmt1), 59 min (rsmL), and 54 min (lolB) (Table 3).

**Table 3:**
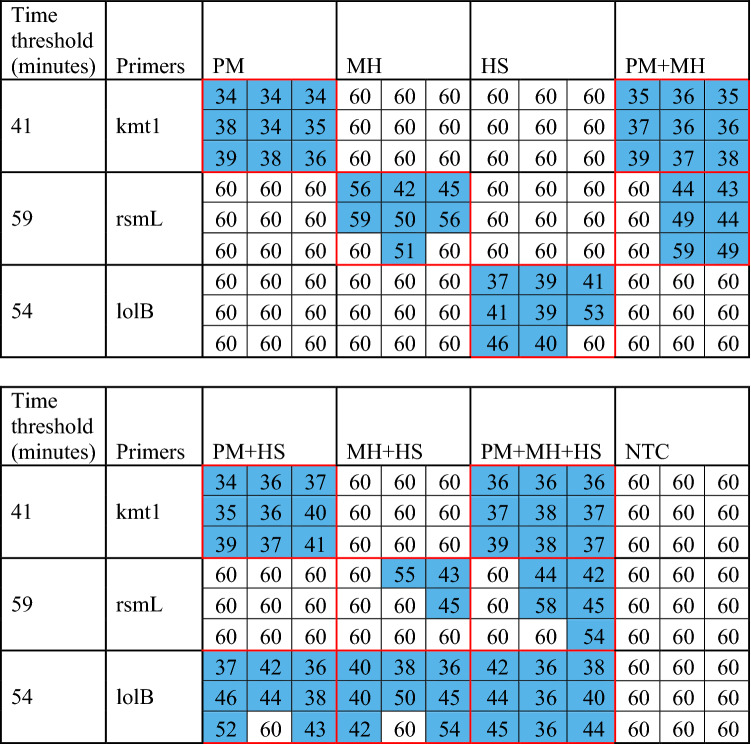
**Loop-mediated isothermal amplification (LAMP) absorbance time thresholds with different combinations of PM, MH, and HS gDNA**

Data illustrating the time (in minutes) obtained from Figure 4 and the samples in which the colorimetric absorbance threshold (430 nm/520 nm) of 3.0 was crossed for each combination of *Pasteurella multocida* (PM), *Mannheimia haemolytica* (MH), and *Histophilus somni* (HS) in the quantitative LAMP (qLAMP) assay shown in Additional file 12. Primer set kmt1 detects PM, rsmL detects MH and lolB detects HS. A value of 60 indicates that the colorimetric absorbance ratio did not reach the 3.0 threshold. The values highlighted in blue are less than or equal to the time threshold for the respective primer set and hence considered positive amplification.

### Colorimetric detection of BRD bacterial pathogens on-farm and in-lab

**Figure 5:**
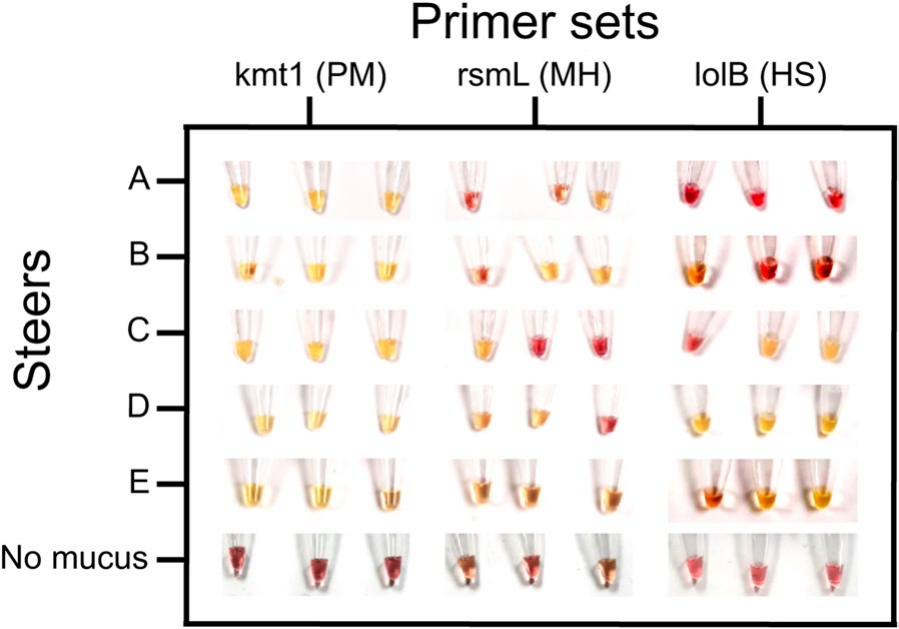
**Colorimetric results of on-farm loop-mediated isothermal amplification (LAMP) detection of bacteria in unprocessed mucus collected from steers.** LAMP reactions were run in polymerase chain reaction (PCR) tubes submerged in water at 65 °C inside a precision cooker. kmt1 detects PM, rsmL detects MH and lolB detects HS. The reactions were run for 60 min. DNA-free water was used as a negative control. Reactions were diluted in water. Images were collected with a Samsung Galaxy A50 and adjusted using the brightness/contrast tool on ImageJ and the white balance tool on Adobe Lightroom. See Additional file 13 for associated PCR results. PM, *Pasteurella multocida*; MH, *Mannheimia haemolytica*; HS, *Histophilus somni*.

**Figure 6:**
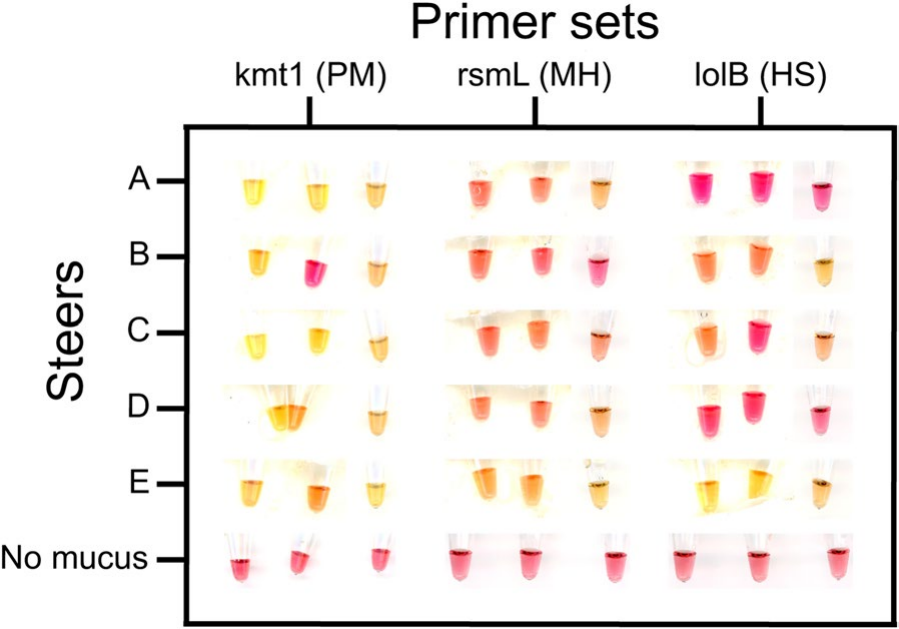
**Colorimetric results of in-lab loop-mediated isothermal amplification (LAMP) detection of bacteria in unprocessed mucus collected from steers.** LAMP reactions were run in PCR tubes submerged in water at 65 °C inside a precision cooker. kmt1 detects PM, rsmL detects MH and lolB detects HS. The reactions were run for 60 min. DNA-free water was used as a negative control. Reactions were diluted in water. Images were collected with the Epson Perfection V800 Photo scanner and adjusted using the brightness/contrast tool on ImageJ and the white balance tool on Adobe Lightroom. See Additional file 13 for associated PCR results. PM, *Pasteurella multocida*; MH, *Mannheimia haemolytica*; HS, *Histophilus somni*.

The reagents for LAMP reactions were prepared in the lab and mucus from different steers was tested for the presence of *P. multocida*, *M. haemolytica*, and *H. somni*. The mucus was initially added on-farm after swabbing the corresponding steer and resuspending the swab contents in 200 µL water. The reactions were then incubated by being submerged in water at 65 °C inside a precision cooker for 60 min (Figure 5). Four days later, the same assay was performed in a lab setting using the same mucus dilutions (Figure 6). The concordance observed between LAMP assays performed on the farm and in the lab varied between primers: kmt1 83.3%, rsmL 66.7%, lolB 66.7%. Although lower than expected, in all the cases (except one) in which discordance was observed, the results were positive on-farm and negative in-lab. Therefore, the lack of consistency is likely due to the instability of the mucus samples (rather than the unreliability of the LAMP assay). These results further accentuate the necessity of field-based testing.

The mucus samples used were also analyzed in a PCR study. Within PCR, *P. multocida* was detected in all steers, *M. haemolytica* was only present in steers D and E, and *H. somni* was detected in steers C, D, and E (Additional file 13). Of the 5 steers, 5 out of 5 of the ones tested for *P. multocida* and *H. somni* showed the same results between on-farm LAMP and PCR. In contrast, only 3 out of 5 of the ones tested for *M. haemolytica* were in agreement. From these results, we can conclude that LAMP is just as reliable as PCR in detecting the presence of pathogens as long as optimal primers are designed.

## Discussion

Since the 2000s [12], LAMP has been a widely used method for the detection of pathogens, including bacterial [18–22] and viral targets [23–28]. While a majority of these efforts have been made using fluorescent dyes, there have been some advances in the detection of pathogens by coupling LAMP reagents with dyes that are sensitive to magnesium [29, 30] or pH [28, 31–33]. These dyes allow visualization of the result with the naked eye. In the assay presented here, we build on the primers previously designed by us [16] and couple them with a pH-sensitive colorimetric reagent: phenol red [28]. The primers were screened through the LOD study. Their analytical sensitivity and specificity were determined by studying their behavior with on-target and off-target DNA mixtures, and the concordance of the reaction results between in-lab and on-farm testing was analyzed (Figure 1).

As seen in previous studies, the Warmstart^®^ LAMP 2 × Master Mix, which contains phenol red, is characterized by its transition from pink to yellow as the LAMP reaction occurs and the pH decreases [28, 34]. Positive and negative results using our selected primers for the detection of *P. multocida*, *M. haemolytica*, *H. somni*, and the pink and yellow distinction can be observed in Figure 2.

While some work has been done to determine the presence of BRD pathogens [16, 35], there is no precedent showing that BRD pathogens can be accurately detected in a field setting. In this paper, Table 1 and Additional file 7 highlight the concordance between conducting the assays on-farm and in-lab. Surprisingly, there is a higher concordance between the on-farm LAMP and in-lab PCR, compared to on-farm and in-lab LAMP. This result suggests that the mucus transportation from the farm to the lab may lead to DNA degradation that leads to false negatives in LAMP reactions (but not in PCR).

In this work, we developed a colorimetric assay for BRD pathogens with the following six advantages: (i) it can be conducted on the farm using a simple consumer-grade water bath, (ii) it provides a visual readout and thus, can be analyzed by the naked eye, (iii) it provides a response within 60 min, (iv) it does not require sample processing (e.g., extraction of nucleic acids), (v) it can detect the pathogens *P. multocida* and *H. somni* with high accuracy (100% and 96%, respectively), and (vi) it utilizes a simple non-invasive nasal swab for sampling.

A major limitation of the current assay is the poor performance of the rsmL primer set for targeting *M. haemolytica.* Even though we performed several screening steps first in our previous work [16] and then in the current work, the primer set had poor performance (accuracy of 79%) mainly due to false negatives. Since the primer set was performing well in the pure *M. haemolytica* sample, we speculate that the drop in performance is due to cross-reactivity with other off-target DNA. We will redesign the primer sets for targeting *M. haemolytica* in future work. Another limitation is the low number of clinical samples tested. Although these numbers are sufficient to demonstrate the feasibility of on-farm visual LAMP, they are not sufficient to demonstrate clinical performance. This study serves as a building block for future larger-scale studies.

We anticipate that due to the simple nature of the assay, it can be coupled to the visual observation of animals for clinical signs and help assess the cause of BRD. The assay can determine whether the *P. multocida* and *H. somni* are present in the animals displaying symptoms. The focus of the current work was on demonstrating the feasibility of conducting a visual molecular assay on the farm (instead of the lab). Only the detection of BRD pathogens is insufficient for clinical diagnosis in BRD since these pathogens could also be present in healthy animals; thus, we did not evaluate diagnostic specificity, sensitivity, and accuracy. With further development, quantification of these BRD pathogens could help distinguish between healthy and sick animals. Once we include more targets (e.g., *Mycoplasma bovis*, viruses, antimicrobial resistance genes) in our assay, it could also help guide the treatment regimen for BRD.

## Supplementary Information

**Additional file 1.Screened primer set sequences developed in** [16]**.**

**Additional file 2. LOD analysis script.** The attached python script, ColorimetricAnalysis.py, was used to analyze primer set screening data. To execute, simply import the file and execute “getPrimerSummer(filename, output)” and provide the file name or path along with the output.xls file name or path. The resulting Excel file will contain two sheets; one containing the final primer scoring and the other containing intermediate calculations for each concentration in each primer set.

**Additional file 3. A top-down thermal Image of precision cooker used for LAMP water bath experiments.** Thermal image of the precision cooker used to heat LAMP reactions. The red cursor indicates the point of the highest temperature (65.3 °C) and corresponds to the color white. The green cursor indicates the point of the lowest temperature (23.6 °C) and corresponds to the color black, which is outside the boundaries of the pressure cooker. The central point is indicated by the white cursor and is 65.2 °C). LAMP reactions were submerged in the water on the right side of the precision cooker.

**Additional file 4. 3D model of the PCR tubes holder. A**. PCR tube holder with 2 hanging parts for convenient placement of the tubes and three sets of eight tubes each. **B**. Slider to cover the tubes from floating in the precision cooker.

**Additional file 5. 3D model of the PCR tubes holder.stl files. A**. PCR tube holder.stl file. **B**. Slider to cover the tubes.stl file. Units are mm.

**Additional file 6. LAMP procedure on the farm. A**. Nasal sample was extracted from a steer. **B**. The extracted mucus on the swab was diluted to 200 µL of water. **C**. 5 µL of resuspended nasal swab solution was used as a sample. **D**. 5 µL of resuspended nasal swab solution were added to pre-prepared colorimetric LAMP tubes with different primer sets. **b**. The tubes were incubated for 60 min at 65 °C inside the precision cooker. Previous experiments ensured the submersion of PCR tubes would not cause inward leaking.

**Additional file 7. On-farm vs. in-lab Hue values.** Hue values from the precision cooker experiment on-farm shown in Figure 5 and in-lab shown in Figure 6. For both LAMP experiments and PCR, cells in blue indicate positive samples and cells in white indicate negative samples. Between farm LAMP and PCR, 2 out of 3 LAMP reactions with the same result as PCR was considered agreement.

**Additional file 8. Limit of detection absorbance values.** Limit of detection table showing selection process for primers for each gene target. The numbers indicate the colorimetric absorbance ratio of absorbance measured at 430 nm to 520 nm. Blue cells correspond to colorimetric absorbance ratios higher than 3.0. Missing data indicates conditions that were not tested.

**Additional file 9. LAMP colorimetric results with PM, MH, and HS gDNA present at 60 min.** Water-suspended DNA extracts of the corresponding gDNA were added to water to generate two-fold serial dilutions (10 000 to 78.125 copies of DNA/reaction). kmt1 detects PM, rsmL detects MH and lolB detects HS. These were added to qLAMP assays with the primer sets being tested for 60 min at 65 °C. Water was used as a negative control. Images were collected using the Epson Perfection V800 Photo scanner, and the background was whitened using the ImageJ brightness/contrast setting. PM: *Pasteurella multocida*, MH: *Mannheimia haemolytica*, HS: *Histophilus somni.*

**Additional file 10. Quantitative results of PM, MH, and HS gDNA present in water.** Water-suspended DNA extracts of the corresponding gDNA were added to water to generate two-fold serial dilutions (10 000 to 78.125 copies of DNA/reaction). kmt1 detects PM, rsmL detects MH and lolB detects HS. These were added to qLAMP assays with the primer sets being tested for 60 min at 65 °C. Absorbance ratios above 3.0 were considered positive and absorbance ratios below 3.0 were considered negative. Water was used as a negative control. For primers lolA, lolB and lppB the data points at minute 59 were excluded due to them being negative values potentially due to instrument error. Each panel has three replicates. PM: *Pasteurella multocida*, MH: *Mannheimia haemolytica*, HS: *Histophilus somni.*

**Additional file 11. LAMP colorimetric results with different combinations of PM, MH, and HS gDNA present in water at 60 min.** Water-suspended DNA extracts at 1,250 copies of DNA per reaction were added to qLAMP assays with the primer sets being tested for 60 min at 65 °C. kmt1 detects PM, rsmL detects MH and lolB detects HS. DNA-free water was used as a negative control. Images were collected using the Epson Perfection V800 Photo scanner, and the background was whitened using the ImageJ brightness/contrast setting. PM: *Pasteurella multocida*, MH: *Mannheimia haemolytica*, HS: *Histophilus somni.*

**Additional file 12. Quantitative results of LAMP detection of different combinations of PM, MH, and HS gDNA present in water at 60 min.** Water-suspended DNA extracts at 1250 copies of DNA per reaction were added to qLAMP assays, with the primer sets being tested for 60 min at 65 °C. kmt1 detects PM, rsmL detects MH and lolB detects HS. Absorbance ratios above 3.0 were considered positive and absorbance ratios below 3.0 were considered negative. DNA-free water was used as a negative control. Each panel has nine replicates. PM: *Pasteurella multocida*, MH: *Mannheimia haemolytica*, HS: *Histophilus somni*.

**Additional file 13. PCR confirmation for 5 steers with 3 different primers corresponding to PM, MH, and HS.** PCR was conducted with the extracted genomic DNA from the mucus obtained from respective steers. PCR was performed using Thermo Fisher Phusion™ High-Fidelity DNA Polymerase (F-530XL). The extracted genomic DNA was used as a template for the PCR reaction. 1% agarose gel was used to run the PCR product with 1kbp DNA ladder (NEB N0468S) as a marker. Expected gene sizes were PM: ompP1 – 1180 bp, MH: iktA – 1932 bp and HS: lppB – 404 bp. PM: *Pasteurella multocida*, MH: *Mannheimia haemolytica*, HS: *Histophilus somni.*

## Data Availability

The data supporting the conclusions of this article are included within the article and its additional files.

## Abbreviations

ADDL: animal disease diagnostic laboratory
BHI: brain–heart infusion
BRD: bovine respiratory disease
dUTP: deoxyuridine triphosphate
ELISA: enzyme-linked immunosorbent assays
FN: false negative
FP: false positive
FPR: false positive rate
HS: *Histophilus somni*
HSV: hue saturation values
LAMP: loop-mediated isothermal amplification
LOD: limit of detection
MH: *Mannheimia haemolytica*
NTC: no-template control
PBS: phosphate buffered saline
PCR: polymerase chain reaction
PM: *Pasteurella multocida*
qLAMP: quantitative loop-mediated isothermal amplification
ROC: receiver operating characteristic
TN: true negative
TP: true positive
TPR: true positive rate
TSB: tryptic soy broth
UDG: uracil DNA glycosylase
