# Supplementary figures and images for: On-farm colorimetric detection of Pasteurella multocida, Mannheimia haemolytica, and Histophilus somni in crude bovine nasal samples
Source: Vet Res. 2021 Oct 2;52:126. doi: 10.1186/s13567-021-00997-9 (PMC8487530; doi:10.1186/s13567-021-00997-9)

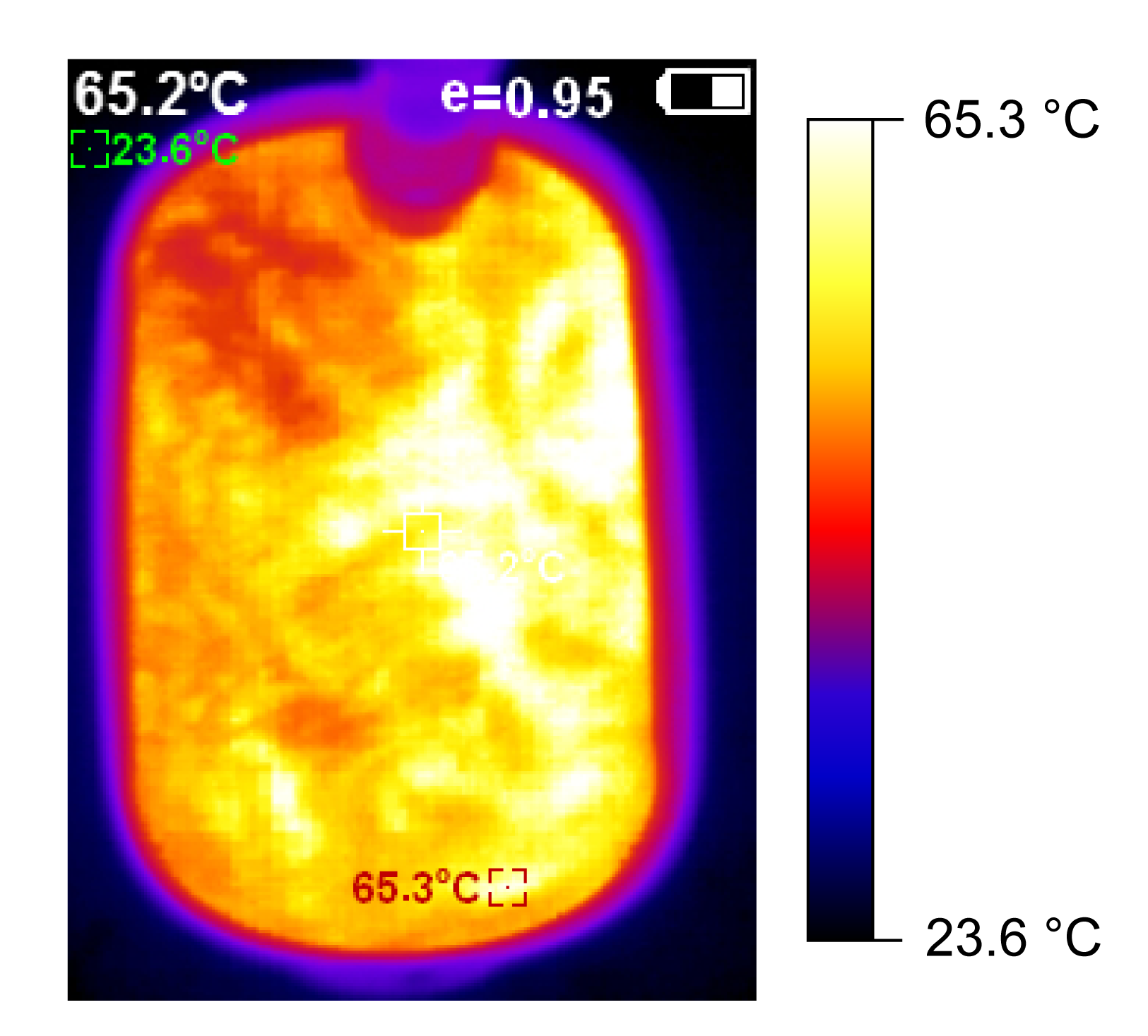

Supplement: Supplementary file 3 — Additional file 3. A top-down thermal Image of precision cooker used for LAMP water bath experiments. Thermal image of the precision cooker used to heat LAMP reactions. The red cursor indicates the point of the highest temperature (65.3 °C) and corresponds to the color white. The green cursor indicates the point of the lowest temperature (23.6 °C) and corresponds to the color black, which is outside the boundaries of the pressure cooker. The central point is indicated by the white cursor and is 65.2 °C). LAMP reactions were submerged in the water on the right side of the precision cooker. [file 13567_2021_997_MOESM3_ESM.png]

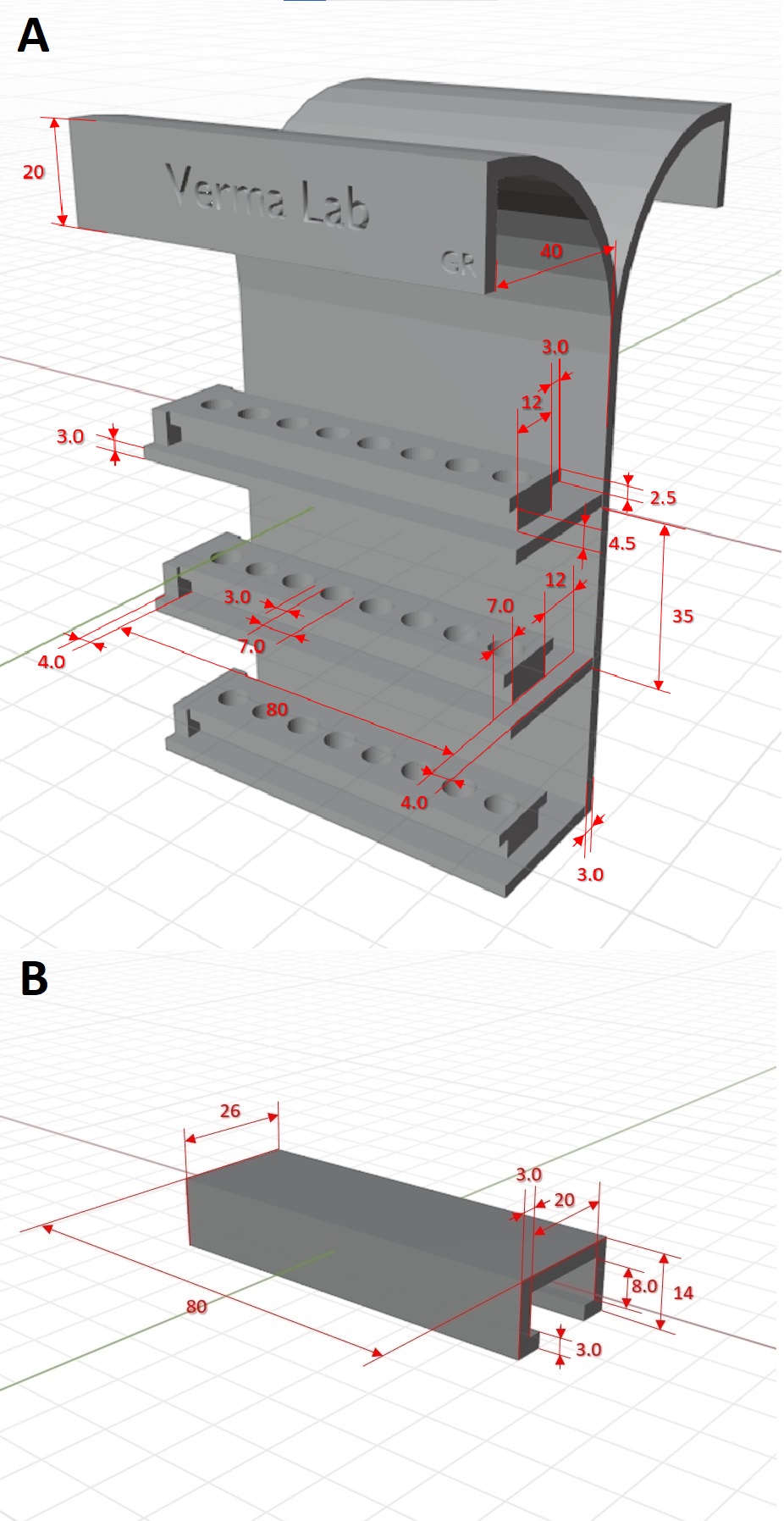

Supplement: Supplementary file 4 — Additional file 4. 3D model of the PCR tubes holder. A. PCR tube holder with 2 hanging parts for convenient placement of the tubes and three sets of eight tubes each. B. Slider to cover the tubes from floating in the precision cooker. [file 13567_2021_997_MOESM4_ESM.jpg]

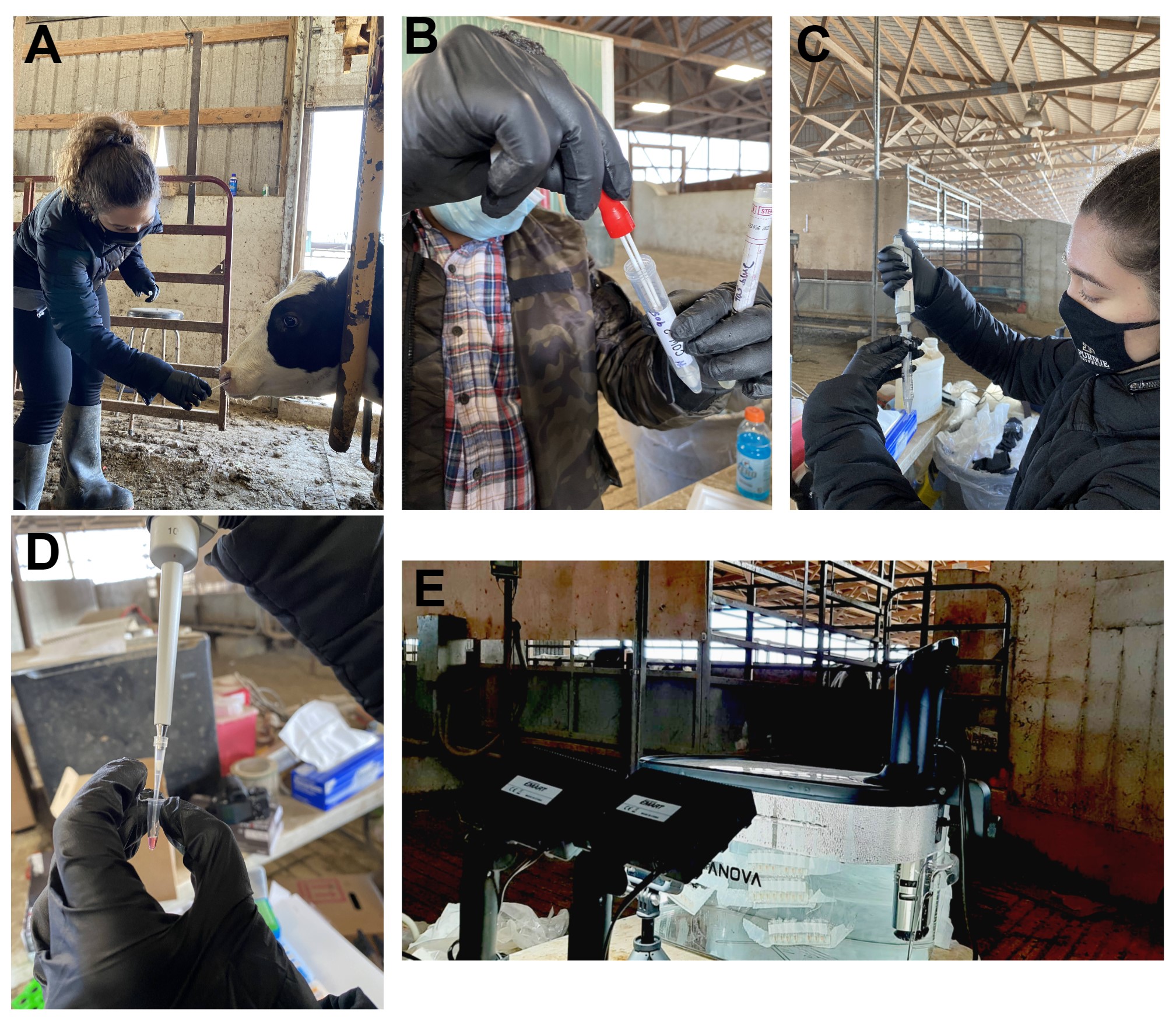

Supplement: Supplementary file 6 — Additional file 6. LAMP procedure on the farm. A. Nasal sample was extracted from a steer. B. The extracted mucus on the swab was diluted to 200 µL of water. C. 5 µL of resuspended nasal swab solution was used as a sample. D. 5 µL of resuspended nasal swab solution were added to pre-prepared colorimetric LAMP tubes with different primer sets. b. The tubes were incubated for 60 min at 65 °C inside the precision cooker. Previous experiments ensured the submersion of PCR tubes would not cause inward leaking. [file 13567_2021_997_MOESM6_ESM.jpg]

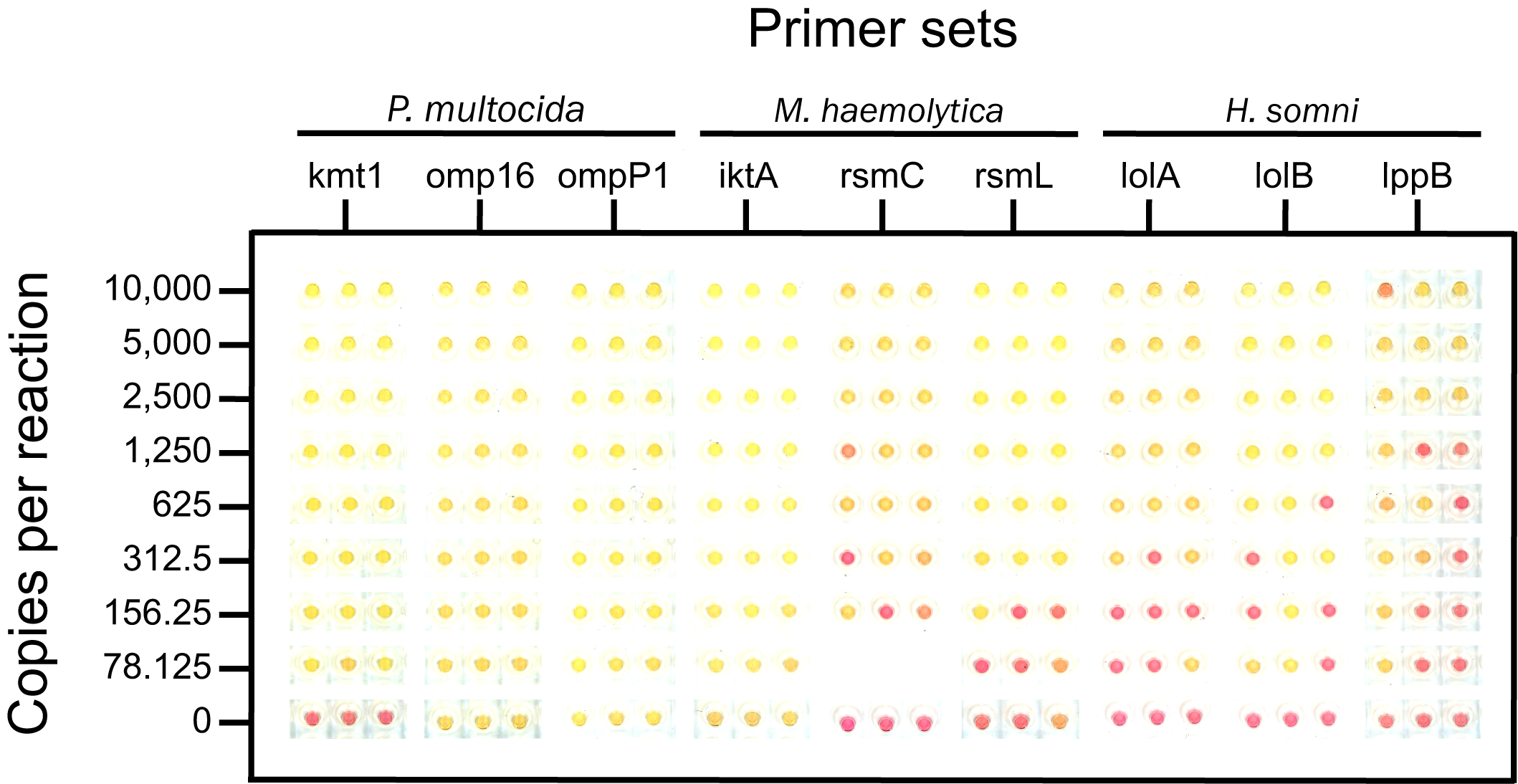

Supplement: Supplementary file 9 — Additional file 9. LAMP colorimetric results with PM, MH, and HS gDNA present at 60 min. Water-suspended DNA extracts of the corresponding gDNA were added to water to generate two-fold serial dilutions (10 000 to 78.125 copies of DNA/reaction). kmt1 detects PM, rsmL detects MH and lolB detects HS. These were added to qLAMP assays with the primer sets being tested for 60 min at 65 °C. Water was used as a negative control. Images were collected using the Epson Perfection V800 Photo scanner, and the background was whitened using the ImageJ brightness/contrast setting. PM: Pasteurella multocida, MH: Mannheimia haemolytica, HS: Histophilus somni. [file 13567_2021_997_MOESM9_ESM.png]

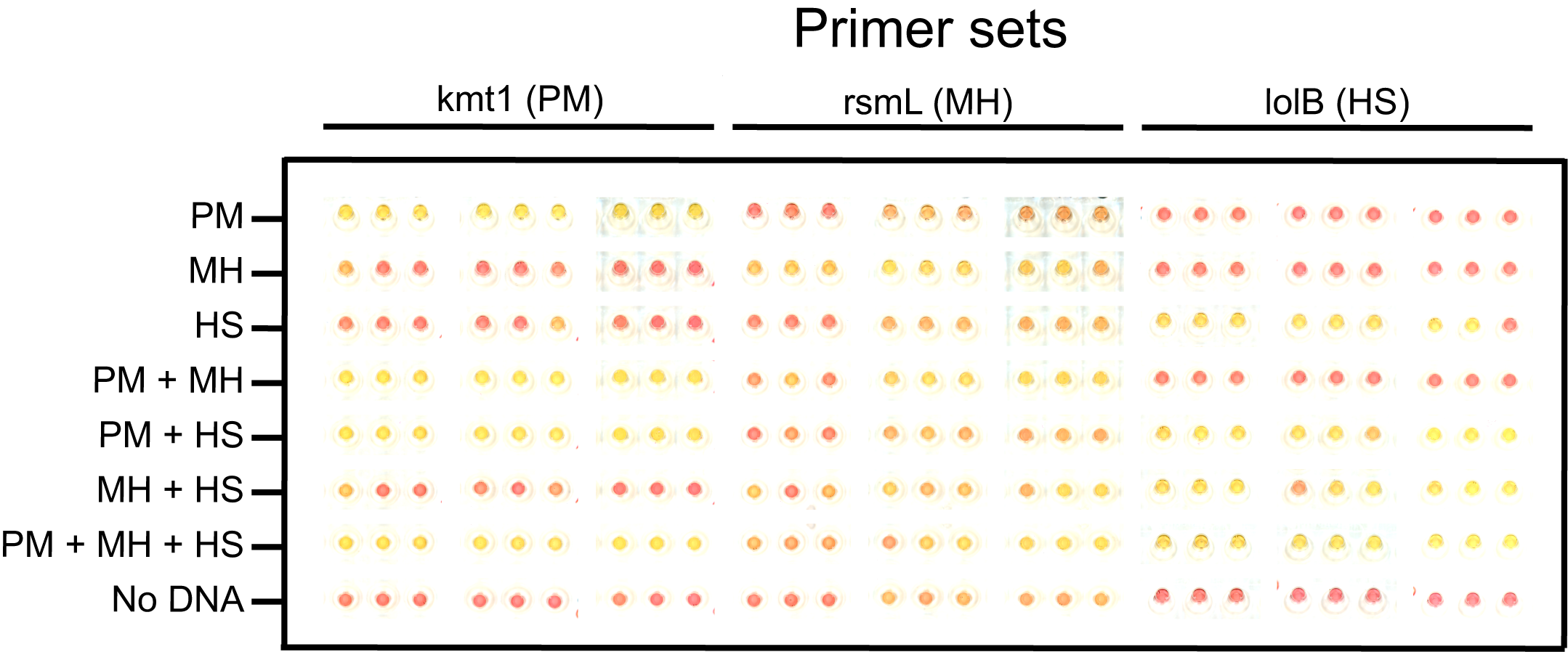

Supplement: Supplementary file 11 — Additional file 11. LAMP colorimetric results with different combinations of PM, MH, and HS gDNA present in water at 60 min. Water-suspended DNA extracts at 1,250 copies of DNA per reaction were added to qLAMP assays with the primer sets being tested for 60 min at 65 °C. kmt1 detects PM, rsmL detects MH and lolB detects HS. DNA-free water was used as a negative control. Images were collected using the Epson Perfection V800 Photo scanner, and the background was whitened using the ImageJ brightness/contrast setting. PM: Pasteurella multocida, MH: Mannheimia haemolytica, HS: Histophilus somni. [file 13567_2021_997_MOESM11_ESM.png]

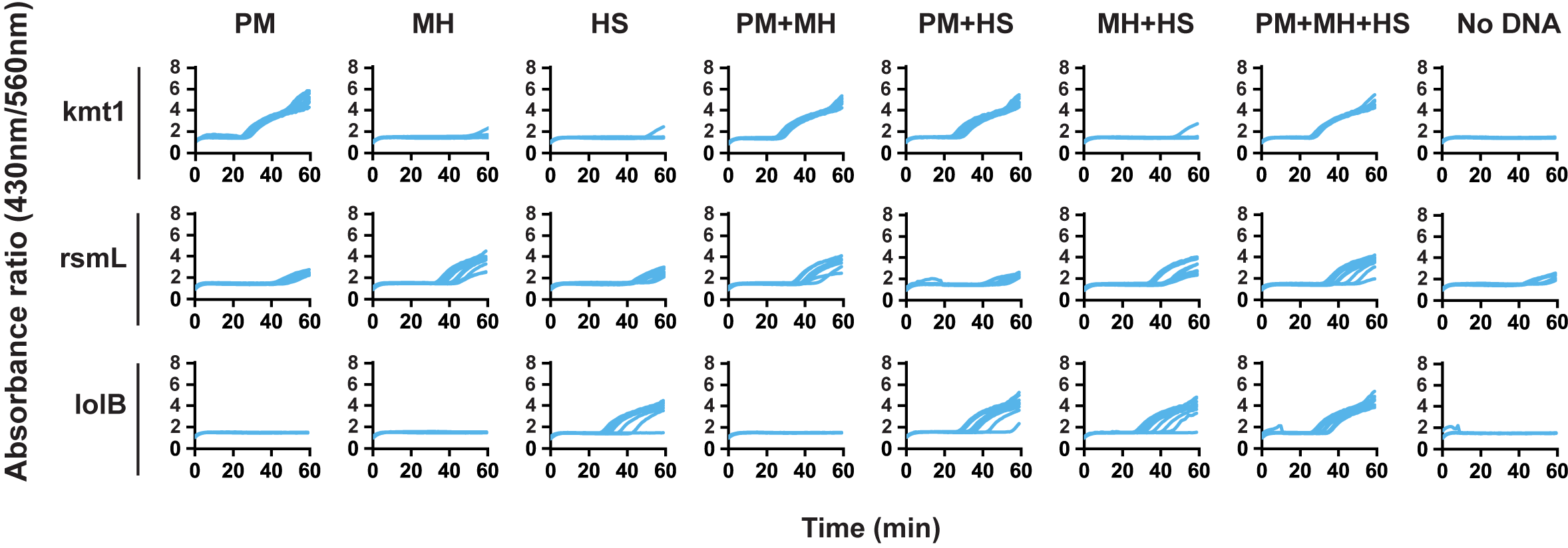

Supplement: Supplementary file 12 — Additional file 12. Quantitative results of LAMP detection of different combinations of PM, MH, and HS gDNA present in water at 60 min. Water-suspended DNA extracts at 1250 copies of DNA per reaction were added to qLAMP assays, with the primer sets being tested for 60 min at 65 °C. kmt1 detects PM, rsmL detects MH and lolB detects HS. Absorbance ratios above 3.0 were considered positive and absorbance ratios below 3.0 were considered negative. DNA-free water was used as a negative control. Each panel has nine replicates. PM: Pasteurella multocida, MH: Mannheimia haemolytica, HS: Histophilus somni. [file 13567_2021_997_MOESM12_ESM.png]

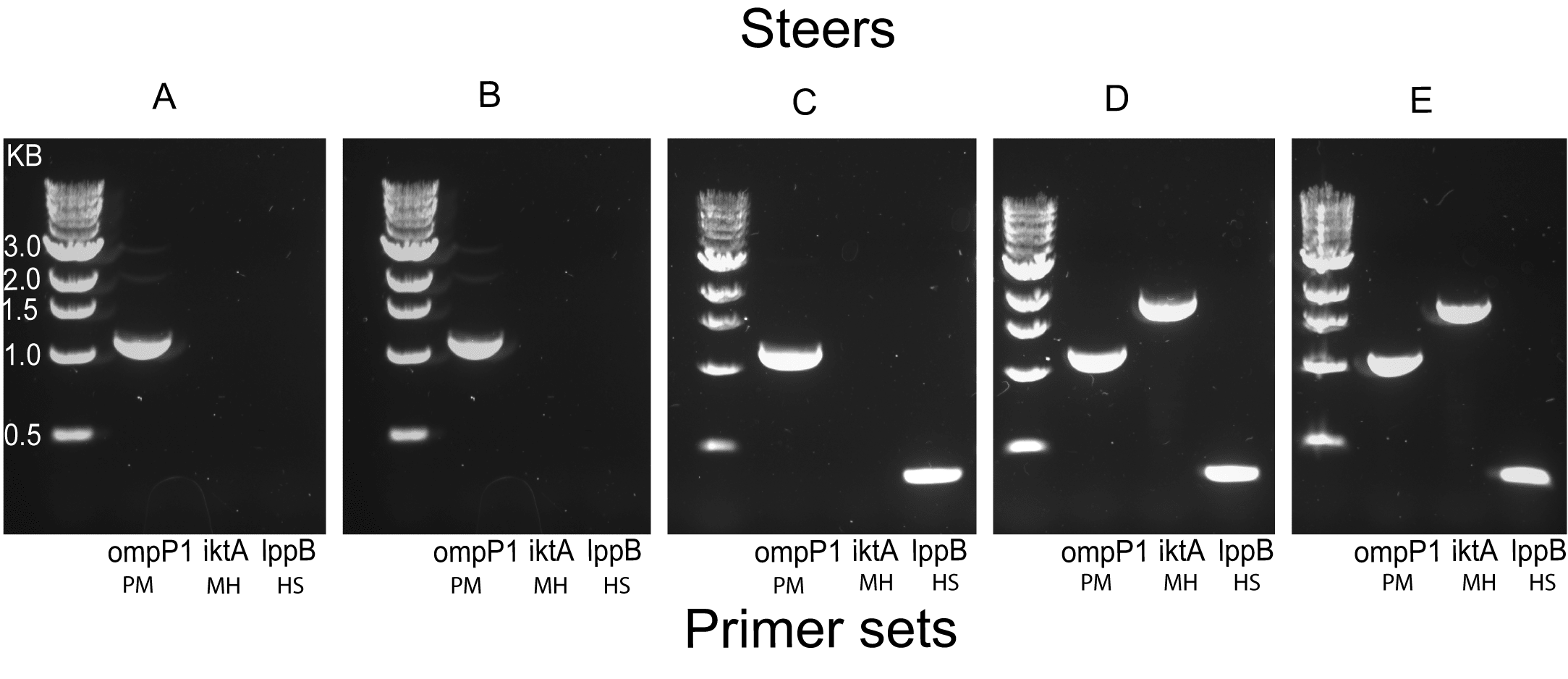

Supplement: Supplementary file 13 — Additional file 13. PCR confirmation for 5 steers with 3 different primers corresponding to PM, MH, and HS. PCR was conducted with the extracted genomic DNA from the mucus obtained from respective steers. PCR was performed using Thermo Fisher Phusion™ High-Fidelity DNA Polymerase (F-530XL). The extracted genomic DNA was used as a template for the PCR reaction. 1% agarose gel was used to run the PCR product with 1kbp DNA ladder (NEB N0468S) as a marker. Expected gene sizes were PM: ompP1 – 1180 bp, MH: iktA – 1932 bp and HS: lppB – 404 bp. PM: Pasteurella multocida, MH: Mannheimia haemolytica, HS: Histophilus somni. [file 13567_2021_997_MOESM13_ESM.png]
